# Evaluating Sparse Inertial Measurement Unit Configurations for Inferring Treadmill Running Motion

**DOI:** 10.3390/s25072105

**Published:** 2025-03-27

**Authors:** Mackenzie N. Pitts, Megan R. Ebers, Cristine E. Agresta, Katherine M. Steele

**Affiliations:** 1 Mechanical Engineering, University of Washington, Seattle, WA 98195, USA; kmsteele@uw.edu; 2 Applied Mathematics, University of Washington, Seattle, WA 98195, USA; 3 Rehabilitation Medicine, University of Washington, Seattle, WA 98195, USA; cagresta@uw.edu

**Keywords:** IMU, machine learning, running, sparse sensing, accelerometer, sampling rate

## Abstract

Inertial measurement units (IMUs) are used to analyze running performance. While leveraging one sensor to estimate kinematic and kinetic variables is common, sparsity limits the number of digital biomarkers that can be evaluated. Shallow recurrent decoder networks (SHRED) can reconstruct a dense set of time-series signals from a single input sensor and have been successful in human mobility applications, highlighting the potential for this algorithm to monitor running. We trained and tested subject-specific SHRED models of nine subjects running on a treadmill to map from one input sensor to the remaining three IMUs. We varied the type of input to reflect experimental parameters that are important in running studies—sensor location, sensor type, sampling rate, and running speed—and compared the error of inferred signals from each input type. Sensor location and type did not impact SHRED inference accuracy, while decreasing the sampling rate affected the accuracy of ankle measurements. All ankle acceleration inferences from these models remained below the minimal detectable change threshold of 12.0 m/s^2^. SHRED models trained and tested at multiple speeds did not accurately infer IMU measurements below this threshold. SHRED may broaden the scope of motion analysis by expanding datasets with fewer sensors.

## 1. Introduction

Measuring biomechanical variables during running can support the evaluation of performance and injury risk. Wearable sensing has grown in popularity as a reliable method for measuring running motion in place of optical motion capture or force plates. Inertial measurement units (IMUs) are one of the most popular wearable sensors for running, especially when leveraging accelerometers and gyroscopes for mobile measurements [1,2]. Importantly, most studies using IMUs for running analysis require only one sensor to estimate a key outcome—for instance, using tibial acceleration to approximate force plate metrics [3]. While measuring with one sensor is convenient, this experimental design produces a limited set of variables to evaluate running motion. Thus, methods to estimate more complex measurements of movement from one or a small set of sensors are a pressing need to support running research and improve performance [1].

The number and type of IMUs used in running studies greatly influences the digital biomarkers that can be estimated and their accuracy. Beyond examining raw segment accelerations, spatiotemporal parameters like cadence or step count can provide valuable insight into athletic performance. Spatiotemporal parameters can be estimated from IMUs placed on the foot, pelvis, or wrist by observing linear acceleration peaks and software-reported metrics, causing variable estimation accuracy depending on the sensor used [3,4]. Using IMUs to estimate joint loads and ground reaction forces is also a common area of research, since linear acceleration is related to force and can provide insight into injury risk and recovery [5,6,7,8]. Leveraging angular velocities measured from gyroscopes, along with linear acceleration, joint [9,10,11], and segment [12] angles, can also be predicted from IMU measurements. These angles are calculated from relative IMU orientations [13], linear mixed effects models [12], or neural networks [14,15]. While these biomechanical variables are useful, multiple sensors are required to accurately measure and collect a sweep of digital biomarkers. For instance, an accelerometer at the thigh is a better predictor of knee loading after anterior cruciate ligament reconstruction [16], but a sensor at the shank or foot provides more accurate estimates of lower-limb kinematics [8,9]. Athletes prefer to wear the minimum number of sensors to reduce the time, weight, and resource burdens [1,3]. A reduced sensor set reduces costs and allows for simple donning but can limit the behaviors that can be observed. Thus, methods that maintain sensing accuracy and portability while enabling access to a dense set of biomechanical data may enhance monitoring of running performance.

Full-body motion estimation using IMUs has gained popularity. Pose reconstruction methods can accurately estimate motion through function optimization [17], neural networks [18,19], or a diffusion model [20]. However, these models require up to six IMUs and rely on known physics or anthropometric data to reconstruct motion. Existing methods that are physics-free estimate digital biomarkers directly [8,9,15], requiring a fixed number of sensors with optimal placements. A motion estimation algorithm that allows for flexibility in experimental design may be useful for running studies due to variations in primary outcome measures and, consequently, sensor selections.

**Figure 1:**
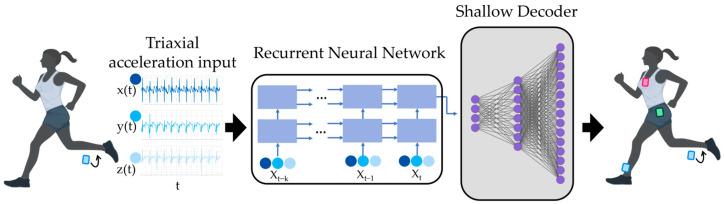
Architecture of a shallow recurrent decoder (SHRED) network. Sparse time-series measurements from an inertial measurement unit (IMU) feed into a recurrent neural network. The encoded signals then feed into a shallow decoder to learn their spatial relationship with the other sensors. The output contains inferred time-series measurements of IMU signals at multiple sensor locations. Modified from [22] with authors’ permission using BioRender.

New developments in machine learning present an opportunity for using a small or sparse set of mobile sensors to reconstruct or infer measurements of multiple sensors (i.e., a full state space). Shallow recurrent decoder networks (SHRED) rely on the time histories of a small set of signals to learn a mapping from a sparse to a dense set of sensors [21]. The architecture features a recurrent neural network—specifically, a long short-term memory network (LSTM)—that learns a temporal relationship among the reduced sensor set, as well as a shallow decoder network to reconstruct or infer the spatial relationship to the unmeasured sensors (Figure 1). These models have outperformed other state reconstruction algorithms that do not account for time histories [22], making SHRED a unique approach for inferring measurements of dynamic tasks. Indeed, SHRED models have been effective for human motion inference by leveraging one IMU sensor to infer IMU signals elsewhere on the body during treadmill walking [23]. Critically, the SHRED architecture is compact [24], so models can quickly be trained and tested on a single subject’s data to improve accuracy; these are termed subject-specific or individualized models. This technique may expand the amount of biomechanical data that are accessible while relying on one sensor, thereby broadening the scope of outcomes available to measure if SHRED is applied to running.

The purpose of this investigation was to evaluate the performance of SHRED for running, with a focus on quantifying how common experimental factors influence the performance of individualized SHRED models. Specifically, we evaluate the inference error—the error between the true measurement and the SHRED-inferred signals—for a single IMU by varying sensor location, sensor type, sampling rate, and running speed. We train distinct SHRED models for each of these experimental parameters to independently evaluate how decisions regarding data collection and model training influence model performance. Other sparse sensing studies rely on an optimal sensor location or type to accurately estimate digital biomarkers [8,9], whereas SHRED model performance is independent of input sensor location, and adding input signals marginally improves accuracy during treadmill walking [22]. Thus, we hypothesized that, when applied to treadmill running, input sensor location and type would not significantly affect the inference of IMU signals. Because SHRED learns a temporal relationship for the input signals during model training, the sampling rate dictates the amount of information that encompasses the time history. Consequently, we hypothesized that reducing the sampling rate would decrease model performance, defined by an increase in inference error. Lastly, we extend the scope of [23] to assess the generalizability of SHRED to extrapolate across multiple running speeds.

## 2. Materials and Methods

### 2.1. Dataset

To rigorously evaluate SHRED for running applications, we required a dataset with multiple known running speeds and multiple IMUs on the lower extremities and upper body. Additionally, we focused on one dataset to facilitate the training of individualized SHRED models rather than merging multiple studies to create a population-based model. We selected an open-source dataset that included ten adult participants (8M/2F, 27.4 ± 4.5 years, 1.76 ± 0.09 m, 69.1 ± 9.9 kg) who completed three 6 min running trials on a treadmill at speeds of 1.8, 2.2, and 2.7 m/s in a random order [25]. This dataset also includes walking trials, which we used to train SHRED models; the results of this training are reported in [23]. One subject did not complete all the running trials, so their data were removed from all analyses. Four triaxial IMUs (Opal V1, APDM Inc., Portland, OR, USA) sampled at 128 Hz were attached to the center of the chest, the left hip, and the lateral side of both ankles. IMUs measured acceleration (range: ±58.8 m/s^2^), angular velocity (±34.9 rad/s), and magnetic field (±600 µT) triaxially, totaling 36 distinct signals across the four sensor locations. For this study, we focused on acceleration and angular velocity signals, as they are commonly used in the literature to estimate digital biomarkers during running [3].

### 2.2. Shallow Recurrent Decoder Parameters

We trained and tested subject-specific SHRED models that map measurements from a single IMU to a dense set of IMU signals using the pyshred package in Python (version 3.11.3). This package has been used in other experiments with tuned hyperparameters [21,22,23,24]. The *shred* function requires an input of the number of sparse signals, as well as the total number of output signals to be learned. Hyperparameters are necessary for defining the neural network architecture. For the *shred* function, these include the following: the number of units in the LSTM latent representation (default: 64), the number of hidden layers in the shallow decoder network (2), the number of nodes in each layer (350, 400), and the dropout rate (0.0). We kept the default settings for these hyperparameters and changed the dropout threshold to 0.1, which supports regularization to avoid overfitting by dropping some nodes during training. To fit the SHRED model for signal inference, training and validation datasets are required. The training set fits the model by using a gradient descent to learn model weights, while the validation set is reserved for monitoring the performance of these weights after each iteration. The partitions for the training, validation, and test sets depend on the experimental parameters and are defined in the proceeding section. The *fit* function within the pyshred package also designates default hyperparameters for training and validation. These include the following: the number of samples batched, shuffled, and evaluated before updating model weights (64); the maximum number of epochs during training (4000); the learning rate of model weights (0.001); and the number of consecutive times the validation error remains consistent before ending training early (5). We only reduced the maximum number of epochs to 500 as we found in preliminary tests that models stopped training before reaching this number.

In this study, a subject’s SHRED models were characterized by altering the type or quantity of the input data. The hyperparameters and output signals remained constant. All models featured a time history of one second. Since the SHRED architecture features an LSTM, this time history represents one second of the sparse input sensor through which a temporal relationship is learned for a current time step.

### 2.3. Experimental Parameters

Multiple configurations of experimental parameters were selected to evaluate how common experimental factors in running studies impact individualized SHRED model performance. Input sensor location, input sensor type, and sampling rate were evaluated by training and testing models on the 2.2 m/s running speed data, selected for convenience. Partitions assigned the first 60% of a trial to the training set, the next 20% to validation, and final 20% to test. We chose this partitioning scheme to reflect the applications of a deployed SHRED model in reconstructing sequential data rather than random samples. Details regarding varied running speeds are presented in Section 2.3.4.

#### 2.3.1. Input Sensor Location

For each subject, we trained three models with distinct input sensor locations. An IMU at the distal tibia and/or lower back are most common in running studies using wearables [3] and a trunk accelerometer is also often used for GPS tracking during running tasks [26,27]. Thus, we selected the right ankle, hip, and chest sensors as proxies for these locations. Since each of these models removed the left ankle as an input location, left ankle measurements were considered “unmeasured” signals that were inferred from each SHRED model. Thus, we leveraged the left ankle output signals across the three models for a statistical comparison of model performance.

#### 2.3.2. Input Sensor Type

Angular velocity measurements are useful for estimating joint angles [9,11,12,13,15], but not all IMUs contain gyroscopes to measure this quantity. To test inference accuracy across signal types, we trained subject-specific SHRED models with different input signals to represent three types of sensors: uniaxial vertical acceleration, triaxial acceleration, and triaxial acceleration and triaxial angular velocity. For this analysis, we focused on evaluating the right ankle IMU as the input data, since this is one of the most common sensor locations in prior running research [3].

#### 2.3.3. Sampling Rate

Sensor battery life and storage depend on sampling rate. We downsampled the data from 128 Hz to 64, 32, and 16 Hz, then trained subject-specific SHRED models for each of these sampling rates. A Fast Fourier Transform for each participant showed that the dominant frequencies for each signal were below 8 Hz, which aligns with the prior literature on inertial signals during running [28] and indicates that aliasing was not a concern when downsampling. We used the right ankle triaxial accelerometer for input.

#### 2.3.4. Running Speed

Individuals run at a range of speeds, so understanding the impact of speed on SHRED accuracy is important for minimizing the number of SHRED models required to infer motion across speeds. First, we trained interpolation models using the slowest (1.8 m/s) and fastest (2.7 m/s) running speeds, then tested these models on the middle speed (2.2 m/s). We also trained extrapolation models on the two slowest speeds and on the fastest speed. Because each of these models evaluated an unseen speed, we maximized variability in the training set while keeping the sequential partitioning scheme. Training, validation, and test data were partitioned by combining data from the two training speeds into one 12 min trial, with the slower speed occurring first. From here, the first 30% and last 30% of the combined trial were assigned to the training set, the centermost 20% was assigned to the test set, and the rest were assigned to validation. In this way, each set contained equal portions of the data from both running speeds. Once trained, data from the unseen third speed were fed to the model, and these inferences were analyzed to evaluate model performance. All models used the right ankle triaxial accelerometer as the input sensor.

### 2.4. Data Analysis

The performance of the individualized SHRED models was evaluated by computing the root mean squared error (RMSE), mean absolute error (MAE), and mean bias error (MBE) between the measured and inferred IMU signals in the test set. Signal directions were labeled either vertical, anteroposterior (AP), or mediolateral (ML), and one-way ANOVAs of RMSE were performed by the directional group for output acceleration and angular velocity. The left ankle was a shared output or unmeasured location for SHRED models with varied input locations, so left ankle RMSE measurements were used for input location ANOVAs and the comparison of model performance. Since only the right ankle sensor was used as input for all other models, the chest, hip, and left ankle signals were evaluated for the remaining parameters. A significance level of a = 0.05 was selected, and paired *t*-tests were applied to all RMSE pairs if an ANOVA resulted in a *p*-value less than 0.05.

## 3. Results

Individualized SHRED models for treadmill running leverage a small set of input signals to infer a dense set of signals at other body segments. To interpret the biomechanical meaning of inferred IMU measurements, we emphasize the error in vertical acceleration at the left ankle as this signal is the closest to axial tibial acceleration within the dataset. Table 1 presents errors aggregated across participants for each of the experiments. RMSE tended to be larger than MAE, suggesting that outliers may be present in signal inference. For fixed-speed models, MBE was close to zero, indicating that models avoided favoring over- or underestimation. Section 3.1 addresses the performance of SHRED models trained and tested with the same speed, and Section 3.2 covers multi-speed models. All errors reported in the text are RMSE. The results for triaxial accelerations and angular velocities at all output sensor locations, along with the Python code used for training and testing SHRED models, are available in the Supplemental Material and at the following link: https://doi.org/10.5281/zenodo.15093318.

### 3.1. Single Speed

#### 3.1.1. Input Sensor Location

**Figure 2:**
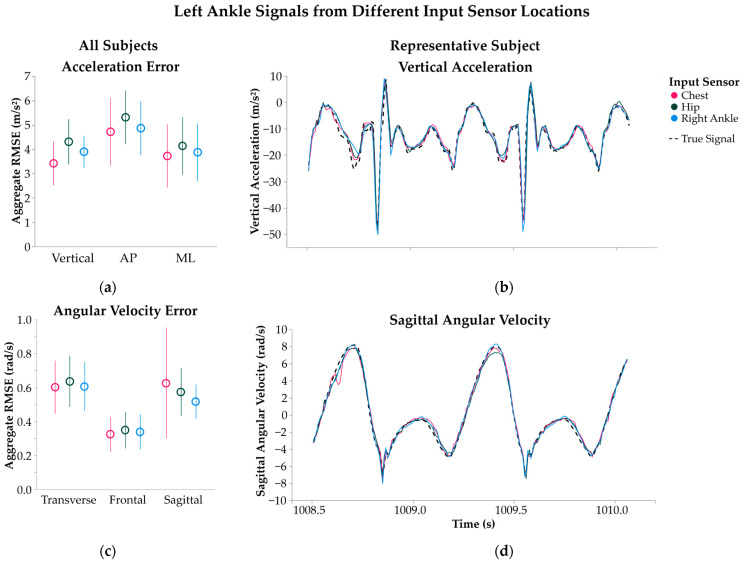
Left ankle signals predicted from individualized SHRED models with input sensor locations at the chest (pink), hip (green), or right ankle (blue). Errors in (**a**) acceleration and (**c**) angular velocity signal inference are measured as root mean squared error (RMSE) and aggregated across all subjects’ models with average ± 1 standard deviation. Signals are further separated by the directions of vertical, anteroposterior (AP), and mediolateral (ML) acceleration and transverse, frontal, and sagittal plane angular velocity. A sample time interval of the inferred left ankle (**b**) vertical acceleration and (**d**) sagittal plane angular velocity is shown for a representative subject’s three SHRED models, along with the true signal measurement (dashed line).

Motion inference error of subject-specific SHRED models was not sensitive to input sensor location. Models with a single input sensor at the chest, hip, and right ankle produced a similar signal inference RMSE (average ± 1 standard deviation) of 3.42 ± 0.90, 4.31 ± 0.93, and 3.90 ± 0.66 m/s^2^, respectively, for left ankle vertical acceleration (Figure 2a, *p* = 0.10). A similar relationship was found in inference errors for the left ankle angular velocity signals (Figure 2c, *p* = 0.87). The effects of sensor location on model error became more distinguishable for output locations that corresponded to the input. For example, a right ankle input sensor resulted in errors of 1.81 ± 0.34, 2.41 ± 0.35, and 1.60 ± 0.34 m/s^2^, when reconstructing vertical, AP, and ML right ankle acceleration signals, while errors in triaxial acceleration at the chest, hip and left ankle averaged 2.16 ± 0.86 2.12 ± 1.13, and 4.21 ± 1.08 m/s^2^, respectively.

#### 3.1.2. Input Sensor Type

**Figure 3:**
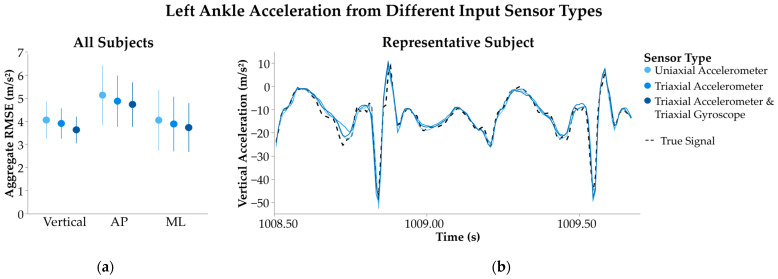
Left ankle acceleration from individualized SHRED models with input sensor types of uniaxial accelerometer (light blue), triaxial accelerometer (blue), or triaxial accelerometer and triaxial gyroscope (dark blue). (**a**) Error of signal inference is measured as root mean squared error (RMSE) and aggregated across all subjects’ models to show average ± 1 standard deviation. Signals are further separated by the vertical, anteroposterior (AP), and mediolateral (ML) directions. (**b**) A sample time interval of the inferred left ankle vertical acceleration is shown for a representative subject’s three SHRED models, along with the true signal measurement (dashed line).

Sensor type had a small effect on SHRED model performance, resulting in more accurate signal inference when more IMU signals were included as the input (Figure 3). For models trained with a uniaxial accelerometer input—representing the sparsest sensor type—left ankle vertical acceleration was estimated with an error of 4.04 ± 0.81 m/s^2^. On the other hand, a triaxial accelerometer and triaxial gyroscope input—the least sparse sensor type—yielded an error of 3.62 ± 0.58 m/s^2^. The differences in model performance for vertical (*p* = 0.43), AP (*p* = 0.75), and ML (*p* = 0.86) signals were not significant.

#### 3.1.3. Sampling Rate

**Figure 4:**
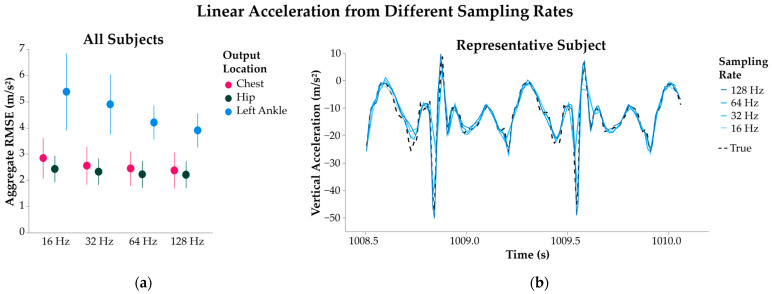
Linear acceleration from individualized SHRED models with sampling rates of 16, 32, 64, and 128 Hz. All models were trained with a right ankle triaxial accelerometer input. (**a**) Error of signal inference is measured as root mean squared error (RMSE) and aggregated across all subjects’ models, shown as average ± 1 standard deviation. Signals are further separated by the output locations of chest, hip, and left ankle. (**b**) A sample time interval of the inferred left ankle vertical acceleration is shown for a representative subject’s four SHRED models with varying sampling rates, along with the true signal measurement (dashed line).

Reducing the sampling rate increased inference error across all output sensor locations; however, significant differences were noted only for signals in the vertical direction (Figure 4). When averaged across all individualized models, downsampling from 128 to 64 Hz yielded an 8.3% change in inferred left ankle vertical acceleration (*p* = 0.011). Reducing the sampling rate from 128 to 16 Hz produced a 38.4% change in inferred left ankle vertical acceleration (*p* = 0.009). Percent changes in left ankle acceleration ranged across the three downsampling ratios from 5.4% to 19.2% for the AP direction and 6.1% to 14.8% for ML, but the inference errors were not significantly different between sampling rates in the same direction. The percent changes in inference accuracy at the hip and chest output locations were not significant in any direction.

### 3.2. Multiple Speeds

**Figure 5:**
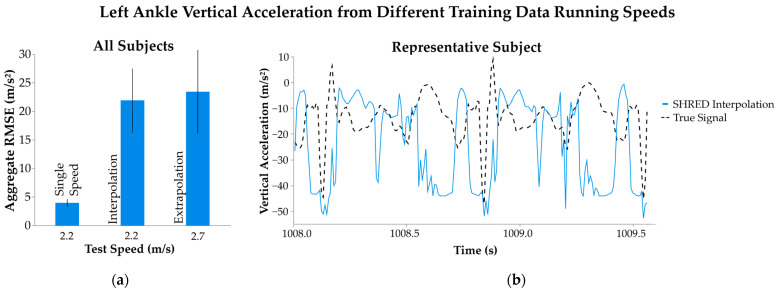
Left ankle acceleration from individualized SHRED models with different training data running speeds. Interpolation models were trained with two speeds and tested with an unseen speed of 2.2 m/s, while extrapolation models were trained using two running speeds and tested with an unseen speed of 2.7 m/s. (**a**) Error of signal inference is measured as root mean squared error (RMSE) and aggregated across all subjects’ models as average ±1 standard deviation. (**b**) A sample time interval of the inferred left ankle vertical acceleration is shown for a representative subject’s interpolation SHRED results, along with the true signal measurement (dashed line).

Individualized SHRED models were inaccurate in reconstructing inertial data for unseen running speeds (Figure 5). Training models on the slowest and fastest speeds yielded an aggregate RMSE of 21.8 ± 5.64 m/s^2^ for vertical left ankle acceleration when testing on the medium running speed. Similarly, the extrapolation models that tested signal inference at the fastest speed produced errors of 23.4 ± 7.32 m/s^2^. This performance greatly contrasts with the inference error of SHRED models that were trained and tested at a single speed, despite having the same input sensor location and type.

## 4. Discussion

In the present study, we trained subject-specific SHRED models to reconstruct a dense set of inertial signals from a sparse input during treadmill running. We quantified the effects of varying sensor location, sensor type, sampling rate, and running speed by training distinct models to illustrate how experimental design may influence model performance. Input sensor location and type had minimal effect on model performance when training and testing models on the same running speed. Conversely, reducing the sampling rate produced greater inference errors. While single-speed SHRED models performed well in inferring unseen sensor trajectories, the performance of SHRED was poor when extended to new running speeds via interpolation or extrapolation. These results demonstrate that individualized SHRED models can reconstruct dense, multimodal running data at fixed speeds, enabling access to unmeasured sensors that could enhance biomechanical assessments in clinic or community settings.

### 4.1. Inference Accuracy

The required accuracy for the reconstruction of IMU signals from a sparse sensor set depends on the application and desired outcomes. Running studies that leverage IMU signals often use the data to predict kinetic, kinematic, or spatiotemporal measures [3,4,5,6,7,8,9,10,11,12,13,14,15,16]. Since one goal of these models is to expand access to data across multiple running sessions using a single sensor, the error at each sensor location can be compared to the minimal important differences or minimal detectable changes (MDC) from the IMU repeatability tests. This comparison can highlight whether the signal inference error falls within the bounds of the variance inherent in the sensor system. Participants in the study presented in Reference [29] completed three 60 s treadmill running trials at 14 kmph while wearing two IMUs on each tibia, repeating the protocol on two consecutive days. For the Delsys Trigno IMU sensor, the between-day MDC for initial peak axial tibial acceleration ranged from 1.22 to 1.41 g (12.0–13.8 m/s^2^). While these values were reported for a different system and at a faster running speed than the conditions in the present study, the errors from the individualized SHRED models in this study were well below these MDCs, suggesting adequate performance for detecting changes at a fixed speed.

#### 4.1.1. Input Sensor Location

Individualized SHRED models accurately inferred inertial signals for various input sensor locations at a fixed speed. Despite the inherent symmetry between the right and left signals, the models trained with a chest or hip input obtained a comparable performance to the right ankle model. This performance aligns with the expectations of SHRED models based on previous applications [22,23]. The ability to achieve a steady performance regardless of the input sensor location makes SHRED valuable across the wide array of IMU systems used in running studies [3], especially when subjects have a preferred location [30]. For circumstances in which measurements at a specific location are a primary outcome—for instance, tibial acceleration to approximate loading—the best practice is to use an input sensor at this location to ensure fidelity, then access the expanded dataset by training a SHRED model.

#### 4.1.2. Input Sensor Type

SHRED model performance was not significantly affected by input sensor type, suggesting that a sparse input can be leveraged across an array of sensor modalities. Given that angular velocity measurements are necessary for estimating kinematics from IMUs during running [9,11,12,13,15], individualized SHRED models can successfully infer angular velocity signals using only the accelerometry input, expanding the types of data that can be studied from wearable sensors. Similarly to the variety in sensor location, diverse sensor types are evident among running studies that use IMUs [1,3], so SHRED may serve as a method to allow for flexibility in experimental design while still connecting to valuable outcomes in the literature.

#### 4.1.3. Sampling Rate

Lowering the sampling rate increased inference error, most significantly at the left ankle. While sampling rates of 16 Hz and 32 Hz are not recommended for analyzing running performance [31], quantifying the error from SHRED models across this range demonstrates the robustness of signal inference during running. Inference error remained below 12.0 m/s^2^ [29] across all sampling rates. Reductions in machine learning performance due to low sampling rates are evident for movement classifiers [32]; however, sampling rates that are too high can negatively impact the estimation accuracy of gait speed for algorithms featuring recurrent neural networks because sequential measurements can appear similar to the model [33]. Thus, the upper limit for sampling rate when training individualized SHRED models for treadmill running remains unknown.

#### 4.1.4. Running Speed

SHRED models were unable to generalize to new running speeds, producing RMSE values significantly above the MDC threshold of 12.0 m/s^2^. The interpolation and extrapolation results demonstrated that SHRED indeed learns the distribution of the training data; limited training data—such as data from only a single individual or from a small number of experimental trials—make it difficult to predict new, complex motions that are not well represented in the training set. As such, the SHRED models of this study are dependent on speed, and thus require a new model for each running speed for use in future experiments. Broadly, when training machine learning models, both the quantity and diversity of the data strengthen their generalizability [34], so additional information will be required to improve the performance of SHRED models across different speeds. These additions may feature more running speeds in the training set, or speed may function as a model input in tandem with acceleration signals.

### 4.2. Significance

Estimations of kinematic or kinetic variables from inertial measurements are often achieved with neural networks, a key feature of SHRED. Much like other motion inference methods using IMUs [9,17,18,19], SHRED models are trained on known input parameters that must remain consistent during testing. The advantage of SHRED is that inference accuracy is independent of the input sensor location and type when a distinct model is trained on these known parameters, which is a limitation of other estimation algorithms [8,9]. Moreover, one sensor can infer the inertial signals at other segment locations. Because studies report that movement classifiers [32,35] and digital biomarker estimation models [8,9,20] improve performance with more than one IMU, accessing additional IMU signals with SHRED can support increased accuracy when estimating digital biomarkers with a single sensor. Additionally, the outputs of SHRED may be used to reconstruct a full state space estimation of unmeasured sensors that could support the training and use of neural networks to infer digital biomarkers from an expanded sensor set.

### 4.3. Limitations

We selected the dataset [25] because the participants wore multiple sensors while running at multiple speeds, providing a broad foundation to rigorously test the validity of individualized SHRED models for human motion inference. However, several features of the dataset limit the immediate translation of SHRED to implementation in future studies and clinical applications. Most importantly, the output of the models were inertial signals, which do not independently characterize digital biomarkers. Existing data reconstruction methods map directly to kinematic and kinetic variables from IMUs [8,9], so there are limited points of comparison for SHRED model performance during running. Therefore, future work should validate whether individualized SHRED models can accurately estimate kinematic and kinetic gait quantities via data expansion given the sparse inertial input, thereby providing more meaningful measures to evaluate running performance. Leveraging the expanded sensor set with new open-source tools for evaluating kinematics and kinetics from IMU data, such as OpenSense [36], presents a promising avenue for future research.

This dataset also contained a small number of sensors, which limits the number of variables that can be estimated from sparse sensing. Additional sensors on the feet, thighs, or wrists may be useful for estimating gait parameters for multiple joints and body segments [9,16]. Lastly, the individualized models were trained and tested on treadmill running data only, so their motion inference accuracy for overground running remains unknown. A key step required to advance toward overground models is improving the performance of models trained and tested on multiple speeds. Specifically, training data should capture the speed range of recreational and elite runners, which can be faster than the maximum speed of 2.7 m/s (6 mph) available in this study. This dataset also had a large difference in running speeds, which may have contributed to the poor interpolation and extrapolation performance. The capacity of SHRED to support interpolation or extrapolation across a more narrow range of speeds remains unknown. Training a SHRED model on multiple subjects to establish a group-based model may also support future research by introducing diverse movements in the training set [23]. Using transfer learning to finetune to an individual could also increase robustness.

Several alternatives exist for partitioning data into training, validation, and test sets. One could reverse the order of sequential intervals such that the test data fall in the first 20% of the trial, the trial could be batched into intervals of equal length and randomly assigned to a set, or all data could be randomly sorted. While this random assignment was successful using SHRED [22], we favored sequential ordering to reflect true implementation of a SHRED model for human motion.

## 5. Conclusions

Subject-specific SHRED models can support running research by expanding access to dense, multimodal time-series data while leveraging the preferred single-sensor experimental design. For example, if during a training study participants run on a treadmill at a specified speed across multiple days or sessions, a SHRED model could be trained on a full sensor set during the first session and then a single sensor could be worn for subsequent sessions to estimate the full sensor set. Selecting input sensor parameters for training remains part of the experimental design. Currently, training studies often only look at pre- and post-outcomes due to the burden of placing sensors, but utilizing SHRED could enable the more robust tracking of changes in biomechanics within and across sessions with a sparse sensor set. The models are robust to varying input sensor location and type, but additional investigation is required to validate their use for multiple running speeds. With the increasing availability of open-source biomechanical datasets, exciting opportunities exist to further test the utility of SHRED, specifically for inferring kinematic and kinetic variables to estimate digital biomarkers. To accelerate these opportunities for future research and support translation, we are sharing an instructional Jupyter Notebook with examples for training and testing individualized SHRED models, available in the Supplementary Material and at the following link: https://github.com/mnp0020/SHRED_Running. This resource will enable researchers to implement, evaluate, and extend these algorithms with their own datasets and applications, including training a population model given a sufficiently large dataset. While the dataset in this study featured participants wearing IMUs while running on a treadmill, the applications of SHRED extend beyond this experimental design for human motion inference. Subjects may be walking, exploring diverse gait patterns, or wearing optical motion capture markers [23]. The value of SHRED lies in its ability to leverage time-series data to learn a relationship across rich, dense datasets, making it a unique tool to study the biomechanics of movement with wearable sensors.

## Figures and Tables

**Table 1:** Aggregate error of inferred left ankle vertical acceleration (m/s^2^, average ± 1SD) from subject-specific SHRED models. RMSE = root mean square error; MAE = mean absolute error; MBE = mean bias error. Shaded conditions indicate parameters that were constant for other experiments.

Experiment	Training Parameter	Running Speed (m/s)	RMSE	MAE	MBE
Input Sensor Location	Chest	2.2	3.4 ± 0.90	2.1 ± 0.52	−0.07 ± 0.27
	Hip		4.3 ± 0.93	2.4 ± 0.44	−0.32 ± 0.33
	Right Ankle		3.9 ± 0.66	2.2 ± 0.38	−0.14 ± 0.23
Input Sensor Type	Uniaxial Accelerometer	2.2	4.0 ± 0.81	2.3 ± 0.43	−0.12 ± 0.17
	Triaxial Accelerometer		3.9 ± 0.66	2.2 ± 0.38	−0.14 ± 0.23
	Triaxial Accelerometer and Triaxial Gyroscope		3.6 ± 0.58	2.1 ± 0.32	−0.16 ± 0.29
Sampling Rate	16 Hz *	2.2	5.4 ± 1.5	2.8 ± 0.54	0.07 ± 0.49
	32 Hz *		4.9 ± 1.1	2.5 ± 0.44	−0.15 ± 0.28
	64 Hz *		4.2 ± 0.65	2.3 ± 0.35	0.01 ± 0.25
	128 Hz		3.9 ± 0.66	2.2 ± 0.38	−0.14 ± 0.23
Multiple Speeds	Interpolation	Train: 1.8, 2.7; Test: 2.2	21.8 ± 5.6	17.1 ± 4.7	9.3 ± 3.8
	Extrapolation	Train: 1.8, 2.2; Test: 2.7	23.4 ± 7.3	18.0 ± 5.7	11.9 ± 6.3

* Statistically different from shaded condition.

## Data Availability

The data presented in this study are available in https://doi.org/10.5281/zenodo.15093318. These data were derived from the following resource, available in the public domain: https://doi.org/10.6084/m9.figshare.7473191.v4 (accessed on 23 November 2023). The pyshred package can be accessed on GitHub at https://github.com/Jan-Williams/pyshred (accessed on 18 August 2023).

## Supplementary Materials

The following supporting information can be downloaded at: https://www.mdpi.com/article/10.3390/s25072105/s1.

## Abbreviations

The following abbreviations are used in this manuscript:

IMU	Inertial measurement unit
SHRED	Shallow recurrent decoder network
LSTM	Long short-term memory network
AP	Anteroposterior
ML	Mediolateral
RMSE	Root mean squared error
MAE	Mean absolute error
MBE	Mean bias error
MDC	Minimal detectable change
